# Synergistic Effects of Some Methoxyflavones Extracted from Rhizome of *Kaempferia parviflora* Combined with Gentamicin against Carbapenem-Resistant Strains of *Klebsiella pneumoniae*, *Pseudomonas aeruginosa*, and *Acinetobacter baumannii*

**DOI:** 10.3390/plants11223128

**Published:** 2022-11-16

**Authors:** Siriwoot Sookkhee, Choompone Sakonwasun, Pitchaya Mungkornasawakul, Phadungkiat Khamnoi, Nitwara Wikan, Wutigri Nimlamool

**Affiliations:** 1 Department of Microbiology, Faculty of Medicine, Chiang Mai University, Chiang Mai 50200, Thailand; 2 Department of Chemistry, Faculty of Science, Chiang Mai University, Chiang Mai 50200, Thailand; 3 Diagnostic Laboratory Unit, Maharaj Nakorn Chiang Mai Hospital, Faculty of Medicine, Chiang Mai University, Chiang Mai 50200, Thailand; 4 Department of Pharmacology, Faculty of Medicine, Chiang Mai University, Chiang Mai 50200, Thailand; 5 Research Center for Development of Local Lanna Rice and Rice Products, Chiang Mai University, Chiang Mai 50200, Thailand

**Keywords:** synergism, antibacterial effect, methoxyflavones, *Kaempferia parviflora*, carbapenem resistance

## Abstract

The present study aimed to investigate the antibacterial activity of ethanolic *Kaempferia parviflora* extracts and the combined effects of the plant’s specific compounds with gentamicin against clinical strains of carbapenem-resistant *Klebsiella pneumoniae*, *Pseudomonas aeruginosa*, and *Acinetobacter baumannii*. The minimal inhibitory concentrations (MIC) of gentamicin and *Kaempferia parviflora* extracts against the tested bacterial strains were determined by using broth microdilution. The combined effects of *Kaempferia parviflora* extract and gentamicin were investigated by using a checkerboard assay and expressed as a fractional inhibitory concentration index (FICI). Crude ethanolic extract of *Kaempferia parviflora* showed the lowest median values of MIC towards the tested isolates (*n* = 10) of these tested bacteria at doses of 64 µg/mL, compared to those of other *Kaempferia extracts*. Among the isolated compounds, only three compounds, namely 3,5,7-trimethoxyflavone, 3,5,7,3′4′-pentamethoxyflavone, and 5,7,4′-trimethoxyflavone, were identified by NMR structural analysis. According to their FICIs, the synergistic effects of gentamicin combined with 3,5,7,3′4′-pentamethoxyflavone were approximately 90%, 90%, and 80% of tested carbapenem-resistant *Klebsiella pneumoniae* (CRKP), *Pseudomonas aeruginosa* (CRPA), and *Acinetobacter baumannii* (CRAB), respectively. The present study concluded that 3,5,7,3′4′-pentamethoxyflavone extracted from *Kaempferia parviflora* potentiated the antibacterial action of gentamicin to combat bacterial resistance against the tested bacteria.

## 1. Introduction

The rapid increase in antibiotic resistance has emerged as a major public health problem of the twenty-first century [1,2]. The antibacterial resistance of Gram-negative bacilli, including carbapenem-resistant *Klebsiella pneumoniae* (CRKP), *Pseudomonas aeruginosa* (CRPA), and *Acinetobacter baumannii* (CRAB) has often been found in hospitals [2]. These carbapenem-resistant strains, which are the cause of nosocomial infection, have recently become resistant to broad-spectrum antibiotics. Infectious diseases, for example bacteremia, severe burns, respiratory, and urinary tract infections caused by these resistant strains have serious negative effects, as their treatment requires higher doses or alteration of antibiotics, possibly prolongs the duration of treatment and hospitalization, causes higher financial costs, and increases mortality rates [3,4]. Recently, many drugs have been used to combat bacterial resistance in addition to gentamicin. Gentamicin is an effective antibiotic and is commonly used for the treatment of patients with a wide range of severe infections caused by Gram-negative bacteria [5]. However, continuing to increase dosages of gentamicin, one of the concentration-dependent killing agents, is not possible due to the narrow therapeutic range, and toxicity may occur.

Alternatively, a combination of this antibiotic and antibacterial plant or herbal extracts or natural compounds has been investigated for the potential therapeutic uses and relatively low side effects, compared with conventional medications [5]. Therapy with herbal extract, alone or in combination with other antibacterial drugs, has several advantages including enhancing antibiotic activity and preventing antibiotic resistance [6]. *Kaempferia parviflora* (KP) is a Thai traditional plant in the *Zingiberaceae* family and *Kaempferia* genus. It is commonly called Thai black ginger or in Thai “Kra-Chai-Dum”. KP has previously been reported to exert several pharmacological effects including antibacterial [7], antifungal [7], antiplasmodial [8], antimycobacterial [8], antipeptic ulcer [9], antiallergic activities [10], and anticancer properties [11,12,13]. Moreover, the essential oils of *Kaempferia* plants have been traditionally used in the treatment of several diseases, for example indigestion, skin diseases, piles, coughs, fever, asthma, and rheumatism. Furthermore, medicinal applications of plants in this genus, such as antimicrobial and larvicidal activity, antioxidant, and anti-inflammatory properties have been reported [14,15,16]. The ethanolic extract of KP rhizome has been widely used in traditional medicine, including for its antibacterial and anticancer activities [7,11]. Methoxyflavones within ethanolic KP (EKP) extract have been shown to exhibit strong anticancer activities against HeLa cervical cancer cells, by suppressing the MAPK and PI3K/Akt signaling pathways stimulated with EGF [13]. EKP extract induced HeLa cell death, via stimulating the intrinsic apoptotic pathway, and via the suppression of crucial molecular signaling at toxic and non-toxic doses of KP, respectively [11]. Flavones have mainly been found in other *Kaempferia* species, but they have also been detected in different plant species [17,18]. Several activities of these compounds have been reported, including antioxidant, antimicrobial, skeleton muscle hypertrophy, and hypolipidemic activities [19,20,21,22]. A previous HPLC study revealed that the major chemical compounds of EKP extract were 5,7,4′-trimethoxyflavone, 3,5,7-trimethoxyflavone, 3,5,7,4′-tetramethoxyflavone, 3,5,7,3′,4′-pentamethoxyflavone, 5-hydroxy-3,7,3′,4′-tetramethoxy flavone, 5-hydroxy-7-methoxyflavone, 5-hydroxy-7,4′-dimethoxyflavone, 5-hydroxy-3,7-dimethoxyflavone, and 5-hydroxy-3,7,4′-trimethoxyflavone; these were identified by comparing their retention times from a chromatogram to those of the standard methoxyflavones [13]. Methoxyflavones possess strong antibacterial activity, as determined by an agar-disc diffusion assay [23]. The antibacterial activity of EKP extract was also found to be active against *Escherichia coli*, *Klebsiella pneumoniae*, *Pseudomonas aeruginosa*, *Staphylococcus aureus*, and *Acinetobacter baumannii*. It can be suggested that some methoxyflavones isolated from EKP extract possess remarkable antibacterial activities against certain bacterial strains. Based on the above, the present study aimed to evaluate the antibacterial activities of crude ethanolic *Kaempferia* extracts and KP compounds alone and in combination with gentamicin against three clinically important bacteria, CRKP, CRPA, and CRAB, isolated from hospitalized patients.

## 2. Results

### 2.1. Minimum Inhibitory Concentrations (MIC) of the Crude Ethanolic Extracts of Kaempferia

**Figure 1:**
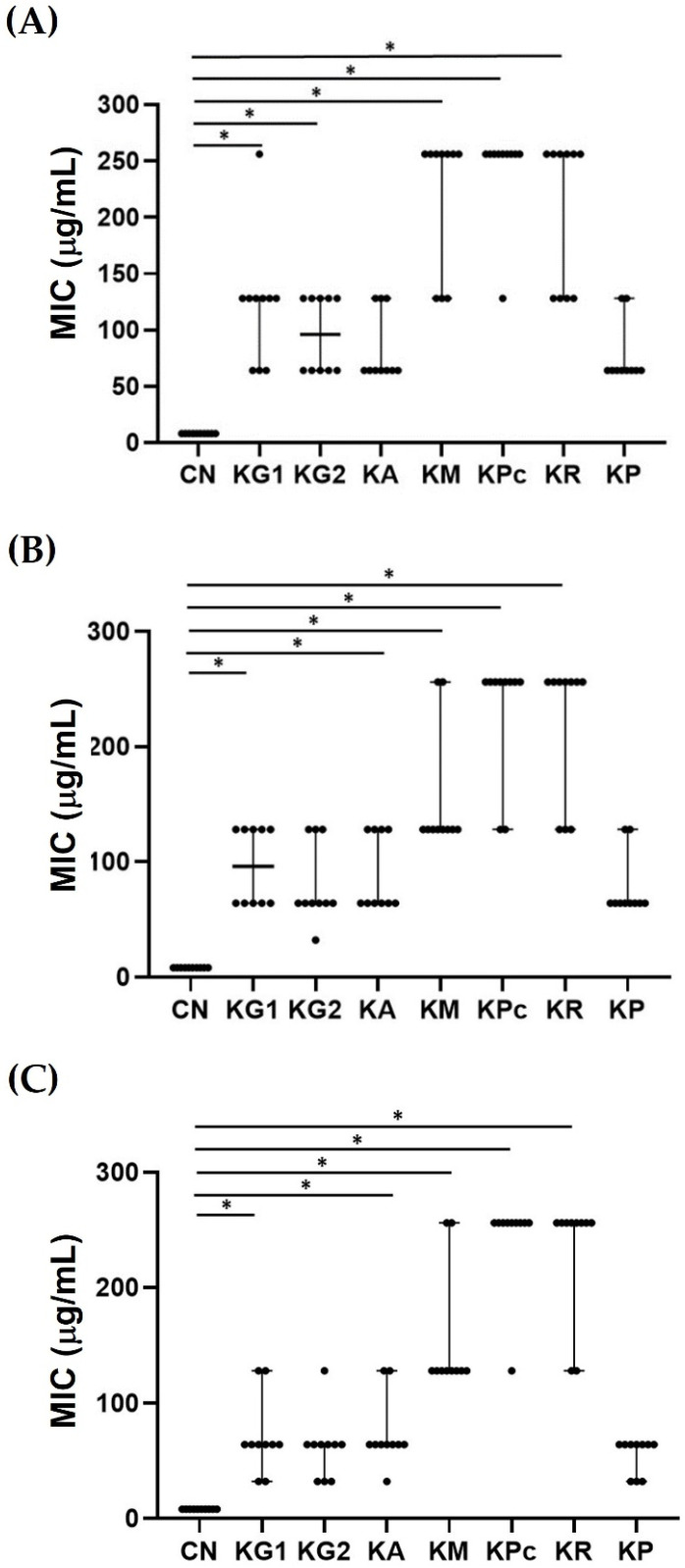
Scatter dot plot showing median values with 95% CI of minimal inhibitory concentration (*n* = 10) of crude *Kaempferia* extracts (*Kaempferia galangal* L. (KG1), *Kaempferia galanga* L. (KG2, *Kaempferia angustifolia* Roscoe (KA), *Kaempferia marginata* Carey (KM), *Kaempferia pulchra* Ridl (KPc), *Kaempferia rotunda* L (KR), and *Kaempferia parviflora* Wall. Ex Baker (KP)), compared to gentamicin (CN) after being separately tested against (**A**) *Klebsiella pneumoniae* (CRKP), (**B**) *Pseudomonas aeruginosa* (CRPA), and *Acinetobacter baumannii* (CRAB) (**C**). *, Significant difference apart from CN at *p* ≤ 0.05.

**Figure 2:**
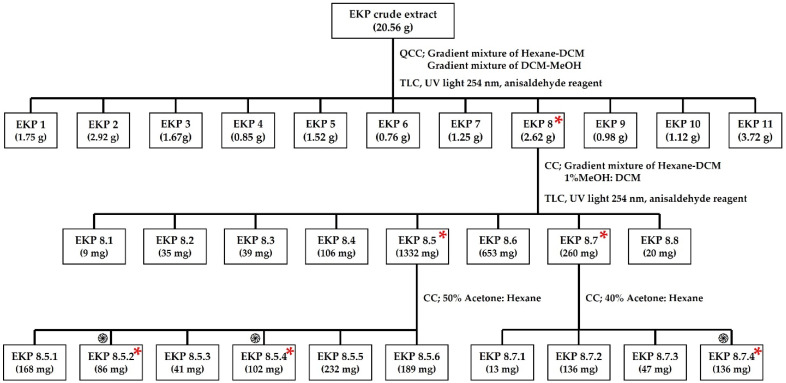
Flow chart presenting the sequential processes for the purification of ethanolic *Kaempferia parviflora* extract (EKP) as described in Section 4.3 in Materials and Methods. Red asterisk (*) indicates the selected EKP that possessed the strongest antibacterial activities (against the tested bacterial strains) in each step of purification. EKP = Ethanolic extract of *K. parviflora*. EKP 8.5.2 = 3,5,7-trimethoxyflavone, EKP 8.5.4 = 3,5,7,3′,4′-pentamethoxyflavone, and EKP 8.7.4 = 5,7,4′-trimethoxyflavone. QCC = Quick column chromatography, CC = Column chromatography, TLC = Thin layer chromatography, UV = Ultraviolet, DCM = Dichloromethane, MeOH = Methanol, and ֍ = Recrystallization.

Among the crude ethanolic extracts of plants in the genus *Kaempferia* (*Kaempferia galangal* L. (KG1), *Kaempferia galanga* L. (KG2, *Kaempferia angustifolia* Roscoe (KA), *Kaempferia marginata* Carey (KM), *Kaempferia pulchra* Ridl (KPc), *Kaempferia rotunda* L (KR), and *Kaempferia parviflora* Wall. Ex Baker (KP)), the antibacterial activities against the carbapenem-resistant tested isolates (*Klebsiella pneumoniae* (CRKP), *Pseudomonas aeruginosa* (CRPA), and *Acinetobacter baumannii* (CRAB)) were only demonstrated in three crude ethanolic *Kaempferia* extracts; these were the crude extracts of *Kaempferia galanga* 2 (KG2), *K. angustifolia* (KA), and *K. parviflora* (KP). In the present investigation, the MICs of crude EKP extract against CRKP, CRPA, and CRAB were 64, 64, and 32 µg/mL, respectively. Additionally, crude EKP extracts showed antibacterial properties against the ATCC strains of these bacteria at 16 µg/mL (data not shown). Only ethanolic *Kaempferia parviflora* extract (EKP) was selected to determine the antibacterial activity of the partial purified and purified extracts (Figure 1A–C). The EKP crude extract was subjected to sequential processes for the purification to select EKP that possessed the strongest antibacterial activities (Figure 2).

### 2.2. MIC of the EKPs after Fractionated Extraction and Purification

**Figure 3:**
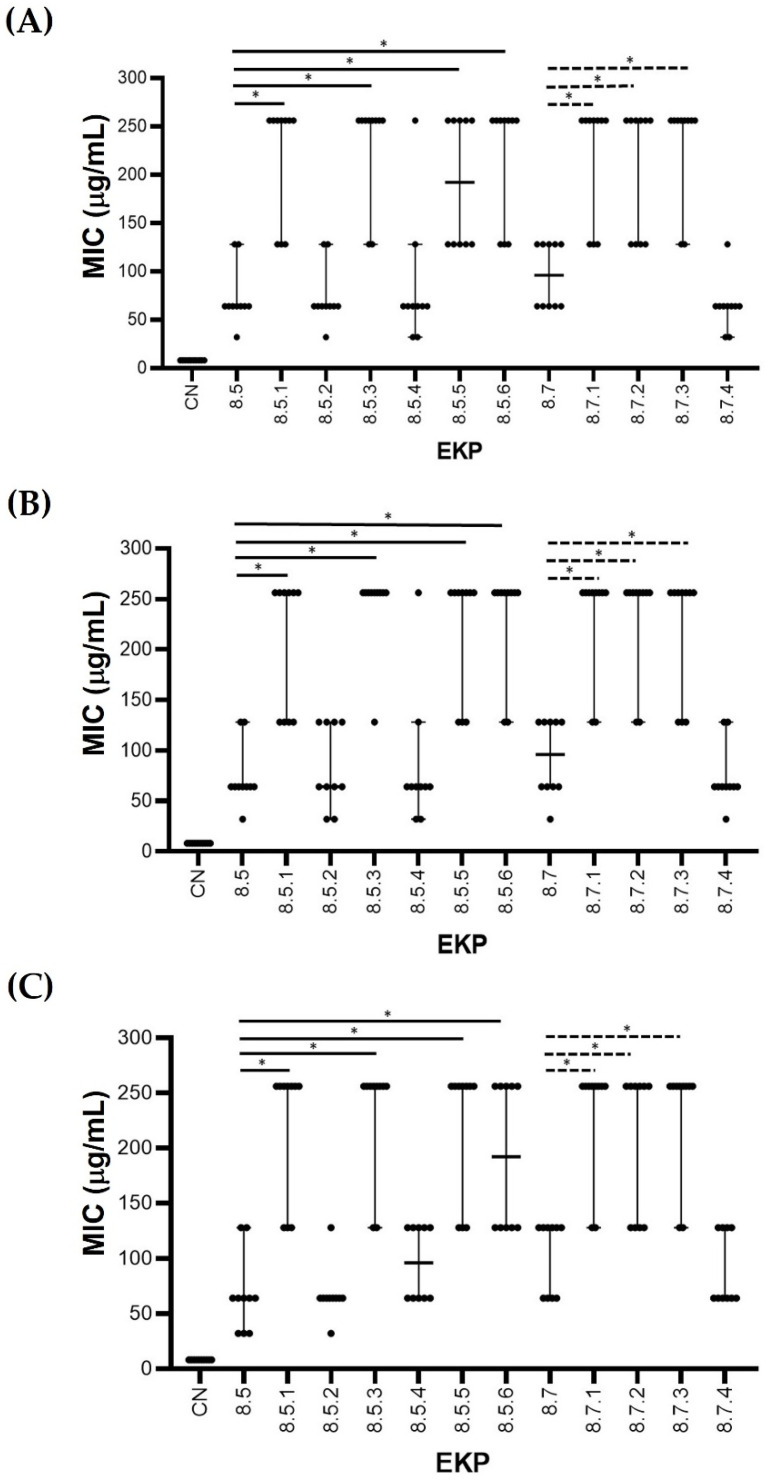
Scatter dot plot showing median values with 95% CI of minimal inhibitory concentration (*n* = 10) of each EKP purified extract compared with EKP8.5 and EKP8.7, which were separately tested with CRKP (**A**), CRPA (**B**), and CRAB (**C**). Gentamicin (CN) was tested as the positive control. *, Significant difference apart from EKP8.5 and EKP8.7 at *p* ≤ 0.05.

In the second step of purification, the antibacterial activity of each EKP extract was also investigated. Antibacterial activities were detected in EKP 8.5 and EKP 8.7 extracts. Further purification steps were performed for these EKP extracts. It was found that only three of 10 EKP extracts, including EKP8.5.2, EKP8.5.4, and EKP8.7.4, showed promising antibacterial effects, compared with their earlier step of extraction, EKP8.5 and EKP8.7, respectively (Figure 3A–C).

### 2.3. Structural Analysis of the Selected Antibacterial EKP Extracts

From the retention time of HPLC and structural investigation by NMR analysis, three methoxyflavones, namely 3,5,7-trimethoxyflavone, 3,5,7,3′,4′-pentamethoxyflavone, and 5,7,4′-trimethoxyflavone, were identically detected to EKP 8.5.2, EKP 8.5.4, and EKP 8.7.4, respectively. The detailed NMR data are shown in Table 1. Additionally, their structures and mass are detailed in Supplementary Figure S1. These methoxyflavones extracted from KP were selected to investigate the combined effects with gentamicin for inhibiting the carbapenem-resistant isolates.

### 2.4. Synergistic Effects against CRKP, CRPA, and CRAB of Three Selected EKP Extracts and Gentamicin

**Figure 4:**
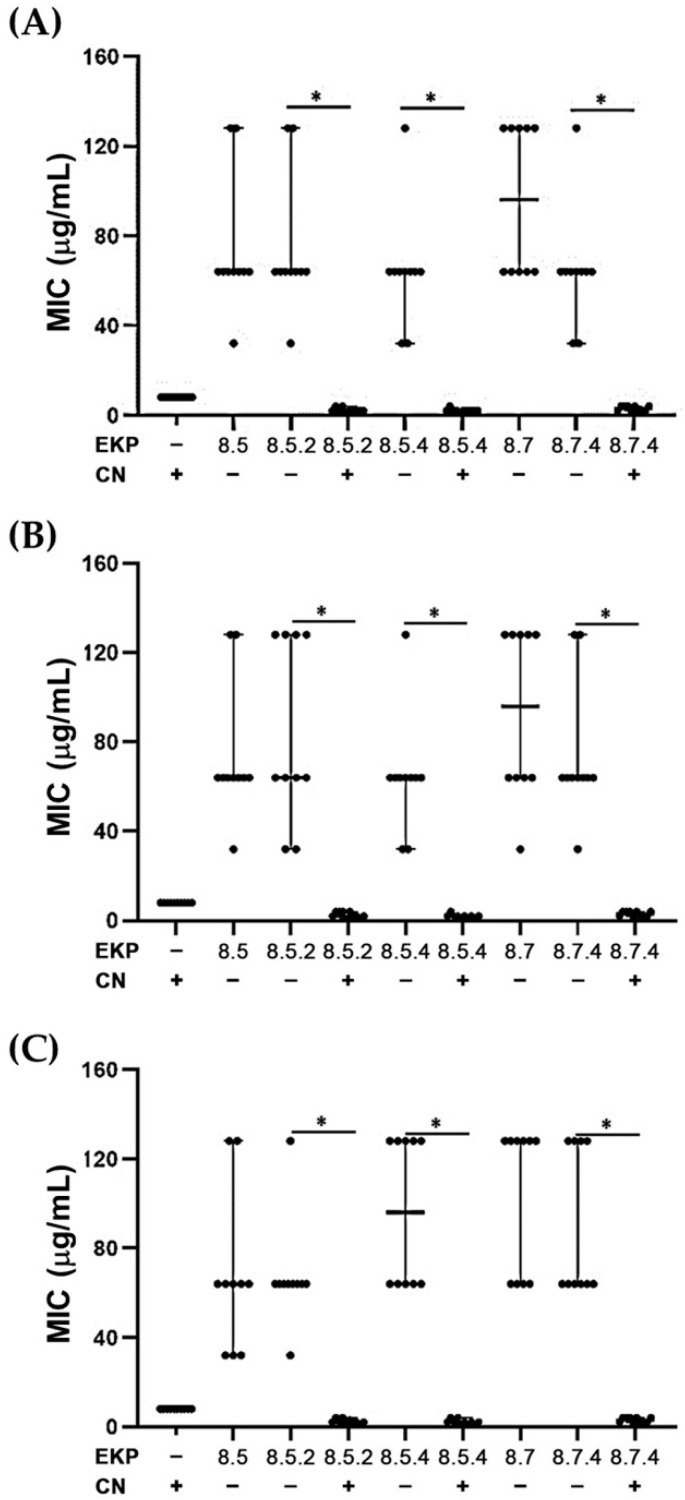
Scatter dot plot showing median values with 95%CI of minimal inhibitory concentration (*n* = 10) of each selected purified EKP extract combined with gentamicin, which was separately tested with (**A**) CRKP, (**B**) CRPA, and (**C**) CRAB. *, Significant difference apart from CN at *p* ≤ 0.05. Solid lines were created after comparing the presence or absence of gentamicin with EKP extract.

According to MIC values of the combinations of EKP 8.5.2, EKP 8.5.4, and EKP 8.7.4 with and without gentamicin against the tested CRKP isolates, the results revealed that significant decreases were detected in the presence of gentamicin against CRKP, as shown in Figure 4A. Figure 4B,C show that the combination was also effective against CRPA (Figure 4B) and CRAB (Figure 4C). These significances are shown as the solid line above the scattered dot of each combination. It is suggested that the antibacterial activities of gentamicin against these tested bacteria could be synergistic with the selected EKP extracts.

**Figure 5:**
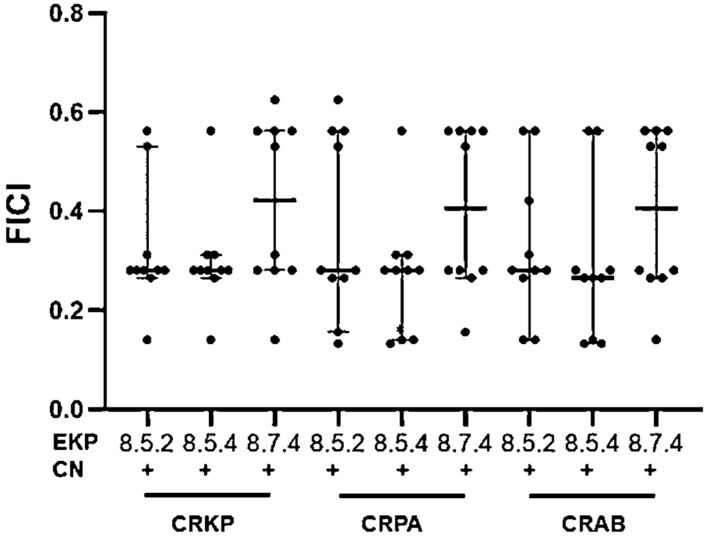
Scatter dot plot showing median values with 95%CI of FICI of the selected purified EKP extracts, EKP 8.5.2, EKP8.5.4, and EKP8.7.4 (*n* = 10) combined with gentamicin (CN), which was separately tested with CRKP, CRPA, and CRAB. *, Significant difference apart from CN at *p* ≤ 0.05 as shown with a solid line.

Figure 5 shows FICI values of the combinations of each EKP purified extract with gentamicin, and suggests that three antibacterial EKP extracts, EKP 8.5.2, and EKP 8.5.4, combined with gentamicin, exerted near to median values of FICI for a synergistic effect against CRKP, CRPA, and CRAB. Higher median values of FICI were shown in the combination of EKP 8.7.4 with gentamicin, after being tested with these tested isolates. This may indicate that the combination of gentamicin with EKP 8.5.2 and EKP 8.5.4 could give strong synergistic effects in all tested bacteria, CRKP, CRPA, and CRAB. This combination may have the potential to use as an antibacterial combination to eradicate the tested carbapenem-resistant bacteria.

The results revealed that EKP 8.5.2, EKP 8.5.4, and EKP 8.7.4 purified extracts showed 80% and 20%, 90% and 10%, and 50% and 50% synergistic and partial synergistic effects to gentamicin against the tested CRKP (*n* = 10), respectively (Table 2, Table 3 and Table 4). As well as CRPA, these three EKP purified extracts demonstrated 60% and 40%, 90% and 10%, and 60% and 40% synergistic and partial synergistic effects to gentamicin against the tested CRPA (*n* = 10), respectively (Table 5, Table 6 and Table 7). The three EKP purified extracts exerted 80% and 20%, 80% and 20%, and 50% and 50% synergistic and partial synergistic effects on gentamicin against the tested CRAB (*n* = 10), respectively (Table 8, Table 9 and Table 10). It should be noted that the highest and lowest synergistic effects were shown in the combination of gentamicin with EKP 8.5.4 or 3,5,7,3′,4′-pentamethoxyflavone, and EKP 8.7.4 or 5,7,4′-trimethoxyflavone.

## 3. Discussion

The increasing incidences of multidrug-resistant nosocomial infections are a serious threat to the present public health care and cause a wide range of problems. Therefore, finding an alternative strategy seems to be important, since discoveries of new antibacterial agents are also limited. Since the median MIC values of EKP were more significant than those of other *Kaempferia* ethanolic extracts, we thus focused mainly on the pure EKP extracts for further comprehensive investigation. Combination therapy of antibiotics with herbal extract could be a novel approach to overcoming nosocomial infections. Meanwhile, increasing values of MICs were demonstrated in the tests of clinical resistant strains. We found that *Kaempferia parviflora* rhizome showed antibacterial activity. This evidence reflected that the clinical strains used in this study, showed high levels of resistance to antibacterial agents. Therefore, in the present study, different combinations of gentamicin and some methoxyflavones from EKP extract were evaluated. EKP extracts that have concentrations of methoxyflavones are more potent in antibacterial activity [24]. In the present investigation, we found that EKP crude extract demonstrated antibacterial activities against CRKP, CRPA, and CRAB. Additionally, it showed antibacterial properties against the ATCC strains of these bacteria. According to our data, the synergistic effects of 3,5,7,3′,4′-pentamethoxyflavone were observed against CRKP, CRPA, and CRAB. Additionally, it was shown that concomitant intake of some EKP extracts may potentiate the antibacterial effect of gentamicin against clinically resistant bacteria, including CRKP, CRPA, and CRAB.

A combination of gentamicin with 3,5,7-trimethoxyflavone, or with 3,5,7,3′,4′-pentamethoxyflavone, and/or with 5,7,4′-trimethoxyflavon, showed a synergistic effect against CRKP, CRPA, and CRAB. This evidence supports the hypothesis that methoxyflavone-containing EKP extracts can increase the antibacterial activities of gentamicin, after lower MICs were detected in these tested isolates. A reduction in the effective dose of gentamicin could be useful in clinical settings, to eliminate its undesired side effects. As seen in Table 3, Table 6 and Table 9, upon combination of gentamicin with 5,7,4′-trimethoxyflavone, partial synergistic effects were mostly observed in these tested isolates. Additionally, in comparison with crude EKP extract, the antibacterial activity of EKP purified extract was more pronounced. The combination of gentamicin with 3,5,7,3′,4′-pentamethoxyflavone enhanced the antibacterial activities of gentamicin. These results support some ethnopharmacological uses of this plant.

The exact mechanism for the different antibacterial activities against the tested strains of these methoxyflavones is still unclear. The antibacterial action of methoxyflavones is not fully understood, but it has been suggested that it is related to the perturbation of the phospholipid/water interface of membranes and consequently increased surface area of the phospholipid head groups in the bilayers [25].

The KP methoxyflavones significantly increased the efficacy of gentamicin against CRKP, CRPA, and CRAB but the mechanism behind this synergistic effect is probably due to the potent anti-efflux activity of gentamicin [24,26]. In addition, it has been shown that herbal extracts that contain flavonoids, alkaloids, tannins, and phenolic compounds, affect the efflux system of bacteria [27].

According to the results shown in Table 2, Table 3, Table 4, Table 5, Table 6, Table 7, Table 8, Table 9 and Table 10, EKP extract, when combined with gentamicin, was effective in inhibiting the growth of bacteria. As mentioned above, these compounds as a part of EKP extract may damage the cell membranes of bacteria and, thereby, enhance the penetration of antibiotics into bacterial cells. This interaction is similar to the interaction of various types of beta-lactams with gentamicin. Some of the KP methoxyflavones’ partial synergistic effect with gentamicin against CRKP could be due to this potent anti-efflux mechanism. However, further study is required to validate this suggestion. Our present observations suggest that 3,5,7,3′,4′-pentamethoxyflavone could be a potential candidate for use along with triple therapy to combat antibiotic resistance and increase the efficacy of treatment of hospitalized patients. Further study is needed to determine the effects of combination of EKP extracts with other antibiotics, such as colistin and fosfomycin, to compare the efficacy of the gentamicin combination against CRKP, CRPA, and CRAB infections. In conclusion, the findings of the present study highlight the advantages of the combination of gentamicin with 3,5,7,3′,4′-pentamethoxyflavone against carbapenem-resistant bacteria and suggest the merit of further investigations and complementary studies.

## 4. Materials and Methods

### 4.1. Plant Materials

Seven *Kaempferia* plants were used in the present study. They are called, in Thai, “Proh-Hom” (*K. galangal* L.; KG1), “Kra-Chae-Chan” (*K. galanga* L.; KG2), “Thao-Nung-Haeng” (*K. angustifolia* Roscoe; KA), “Thor-Ra-Nee-Yen” (*K. marginata* Carey; KM), “Nok-Khum” (*K. pulchra* Ridl.; KPc), “How-Non” (*K. rotunda* L.; KR), and “Kra-Chai-Dum” (*K. parviflora* Wall. Ex Baker; KP). These rhizomes were harvested from the CMU-RSPG *Kaempferia* planting at Chiang Dao, Chiang Mai, Thailand with the voucher specimen numbers R-CMUKG001, R-CMUKG002, R-CMUKA001, R-CMUKM002, R-CMUKPC001, R-CMUKR004, and R-CMUKP002, respectively. The identification was authenticated by Dr. Angkana Inta, and the plant specimens were deposited at the herbarium of the Department of Biology, Faculty of Science, Chiang Mai University, Thailand.

### 4.2. Ethanolic Extraction of Kaempferia Rhizomes

The preparation of the crude extract was performed exactly as reported in the previous study [11]. Briefly, the rhizomes were cleaned, air-dried at room temperature, and ground into small pieces. The dried rhizomes of each species (2 kg) were extracted with 95% ethanol at room temperature (3 × 4 L) for three days. The ethanolic extract was filtered before being concentrated. The extraction process yielded residues of 8–15% dry weight of rhizomes for ethanolic extraction using a rotary evaporator, then lyophilized and kept in an air-tight, light protected container. One thousand and twenty-four µg of each ethanolic crude extract was dissolved in 1 mL of sterilized Mueller Hinton Broth (MHB; Oxoid, Hampshire, UK) containing 0.2% DMSO (Sigma-Aldrich, Saint Louis, MO, USA) to make a stock solution of 1024 µg/mL prior to each assay. Each experiment was performed by using three independent batches.

### 4.3. Fractionated Purification of Ethanolic KP Extracts (EKP)

EKP extracts were purified as shown in Figure 2. All EKPs in each step of purification were evaluated for their antibacterial activities against the tested bacteria, selecting the EKP that possessed the strongest activities to further undergo the purification step, until the purified extracts were harvested. The EKP crude extracts were fractionated by quick column chromatography (QCC) using a silica gel, followed by elution with a gradient mixture of *n*-hexane/DCM (100:0 to 0:100) and DCM/MeOH (100:0 to 50:50) to yield eleven fractions of ethanolic *Kaempferia parviflora* extract (EKP 1 to EKP 11), as previously described [28]. Briefly, fraction 8 (EKP 8, 2.62 g) was fractionated by silica gel column chromatography (CC), using a gradient mixture of *n*-hexane/DCM (100:0 to 0:100) and 1% MeOH/DCM to yield eight fractions (EKP 8.1 to EKP 8.8). Fraction EKP 8.5 (1332 mg) was chromatographed over silica gel using 50% acetone/*n*-hexane as an eluent to yield six fractions (EKP 8.5.1 to EKP 8.5.6). Furthermore, fraction EKP 8.7 (259 mg) was subjected to a silica gel CC using 40% acetone/*n*-hexane to yield four fractions (EKP 8.7.1 to EKP 8.7.4). Fractions EKP 8.5.2, EKP 8.5.4, and EKP 8.7.4 were recrystallized with MeOH to give pure compounds as yellow needle (86 mg), colorless needle (102 mg), and powder (136 mg), respectively. These pure compounds were identified using ^1^H-NMR with comparison with those previously reported for the compounds [28]. All fractions were detected using thin layer chromatography (TLC) under UV light 254 nm and anisaldehyde reagent.

### 4.4. High-Performance Liquid Chromatograph (HPLC) Analysis of EKP Purified Extracts

Each EKP purified extract was identified by high-performance liquid chromatograph (HPLC) and compared with nine standard methoxyflavones. These standard compounds comprised 3,5,7,3′,4′-pentamethoxyflavone (S1), 5,7,4′-trimethoxyflavone (S2), 3,5,7-trimethoxyflavone (S3), 3,5,7,4′-tetramethoxyflavone (S4), 5-hydroxy-3,7,3′4′-tetramethoxyflavone (S5), 5-hydroxy-7-methoxyflavone (S6), 5-hydroxy-7,4′-dimethoxyflavone (S7), 5-hydroxy-3,7-dimethoxyflavone (S8), and 5-hydroxy-3,7,4′-trimethoxyflavone (S9), and were obtained from the Eco-friendly Product Research Laboratory (EfPRL), Department of Chemistry, Faculty of Science, Chiang Mai University, Chiang Mai, Thailand. HPLC analysis of standard compounds was performed on an Agilent 1100 series HPLC instrument equipped with a UV detector (Agilent Technologies, Santa Clara, CA, USA) used for quantification. The separation was carried out on an Ascentis^®^ C18 column (250 mm * 4.6 mm, internal diameter 5 µm; Hypersil^TM^, Sigma-Aldrich, Saint Louis, MO, USA). The mobile phase was methanol/0.5% acetic acid (65:35 *v*/*v*), with operation at a flow rate of 1.0 mL/min as previously described [28]. The injection volume was 20 µL and at least three repetitive injections were performed for the sample and standards. The detection was set at 230, 280, and 355 nm.

### 4.5. Microorganisms

The tested bacteria used in this study were ten carbapenem-resistant isolates of *Klebsiella pneumoniae* (CRKP), of *Pseudomonas aeruginosa* (CRPA), and of *Acinetobacter baumannii* (CRAB), which were provided from the culture plates of nosocomial-infected specimens at the Diagnostic Laboratory Unit, Maharaj Nakorn Chiang Mai Hospital, Faculty of Medicine, Chiang Mai University, Chiang Mai, Thailand. Each isolate was subcultured on Mueller Hinton Agar (MHA; Oxoid, Hampshire, England) and incubated at 37 °C. Their identifications were confirmed by using Vitek-MS apparatus (BioMérieux, Marcy l’Etoile, France) and their antibiotic susceptibilities were investigated by the use of Vitek-2 apparatus (BioMérieux, Marcy l’Etoile, France) at the Diagnostic Laboratory Unit of Maharaj Nakorn Chiang Mai Hospital, Faculty of Medicine, Chiang Mai University, Chiang Mai, Thailand. The carbapenem resistance of the selected isolates was confirmed by determining their MICs toward meropenem, the representative drug of carbapenem. Their MICs toward gentamicin were also evaluated by broth microdilution assay, using VITEK-2 apparatus. According to CLSI guidelines [29] on the MIC breakpoint for meropenem against *K. pneumoniae*, *P. aeruginosa*, and *A. baumannii,* they should be considered resistant if MIC is >4, >8, and >8 µg/mL, respectively. The MIC breakpoint for gentamicin against Enterobactericeae, *P. aeruginosa*, and *A. baumannii* should be considered as susceptible at ≤4 µg/mL, intermediate resistant at 8 µg/mL and resistant at ≥16 µg/mL. Ten isolates of CRKP, CRPA, and CRAB that showed meropenem-resistant, gentamicin-intermediate-resistant, and colistin-susceptible patterns were selected to use as the tested isolates in the present study.

### 4.6. Determination of the Minimal Inhibitory Concentration (MIC)

A broth microdilution assay was performed to determine the minimal inhibitory concentration (MIC) of each tested extract. Briefly, the stock solution of each extract was 2-fold serially diluted with MHB to a total of ten concentrations in 96-well microtiter plates (Corning™, Corning Inc., Salt Lake City, UT, USA). The highest final concentration of the tested EKP extract was 256 µg/mL. Each purified colony of the tested bacterial isolate was inoculated into Mueller Hinton Broth (MHB) and the bacterial suspension was adjusted to 0.5 McFarland standard turbidity [29,30]. Following a 20 h incubation, each well was optically observed for growth or complete inhibition of growth. The MIC was then recorded. Each tested compound was evaluated in triplicate. DMSO diluted in MHB (0.2%) and colistin sulfate were used as the negative and positive controls, respectively.

### 4.7. Determination of Synergistic Effects between each EKP Extract and Gentamicin

Standard checkerboard assays were performed, as described previously [31,32], to evaluate the combined effect of gentamicin and the tested EKP. Fifty µL of EKP solution dissolved with 0.2% DMSO in MHB were serially diluted with MHB along the vertical axis of rows 1–7 (top and bottom: highest and lowest final concentrations were 256 and 4 µg/mL, respectively). In row 8, the two-fold serially diluted EKP extract was not put into the wells, therefore, this row showed only the action of gentamicin, and was called “gentamicin alone”. Fifty microliters of the serial two-fold dilutions of gentamicin (Sigma-Aldrich, Saint Louis, MO) in MHB was put into each well along the horizontal axis of 96-well microtiter plates in columns 1–9 (left and right: highest and lowest final concentrations were 256 and 1 µg/mL, respectively). In the tenth right column of the 96-well plate, the two-fold serially diluted gentamicin was not put into the wells, therefore, this column showed only the action of EKP and was called “EKP alone”. In the eleventh right column of the 96-well plate, 50 µL of MHB and 50 µL of MHB supplemented with 0.2% DMSO was placed into each well, as the negative control, and was called an “untreated well”. These tested isolates confirmed the susceptibility by determining MIC toward colistin sulfate. In the twelfth right column of the 96-well plate, 50 µL of colistin solution and 50 µL of MHB supplemented with 0.2% DMSO were placed into each well as the positive control. From an overnight culture, a suspension was prepared in a MHB solution that corresponded to 0.5 McFarland turbidity. Then, 0.1 mL of this suspension was dissolved in 9.9 mL of MHB, and eventually a solution was obtained—inoculum—containing approximately 5 × 10^5^ CFU/mL at the final bacterial concentration [33]. After that, 100 µL of inoculum was administered into each well of the 96-well microdilution plates. The inoculated microplates were incubated at 37 °C for 24 h. After 24 h, MIC is defined as the lowest concentration that inhibits the growth of the test microorganism [34]. All experiments were performed in triplicate. The fractional inhibitory concentration index (FICI) value was used to assess whether synergism, indifference or antagonism occurred following the combination effects of gentamicin and EKP extract, employing the following formula [35].

FIC of gentamicin = (MIC of gentamicin in combination)/(MIC of gentamicin alone)

FIC of EKP extract = (MIC of EKP extract in combination)/(MIC of EKP extract alone)

FICI of the combination was FIC of gentamicin + FIC of EKP extract.

The combined effect was evaluated based on the following criteria:

FICI ≤ 0.50 denoting synergism;

0.50 < FICI ≤ 0.75 denoting partial synergy;

0.75 < FICI ≤ 1 denoting an additive effect;

1 < FICI ≤ 4 denoting indifference or no interaction

and FICI > 4 denoting antagonism [36].

### 4.8. Statistical Analysis

MICs were non-parametrically analyzed and a scattered dot plot of median value with a 95% confidence interval (CI) was created by using GraphPad Prism. Differences were considered statistically significant if the results of a Kruskal–Wallis test with multiple comparisons showed a *p*-value ≤ 0.05.

## Figures and Tables

**Table 1:** ^1^H NMR data (CDCl_3_, 400 MHz) of 3,5,7-trimethoxyflavone (EKP 8.5.2), 3,5,7,3′,4′-pentamethoxyflavone (EKP 8.5.4), and 5,7,4′-trimethoxyflavone (EKP 8.7.4).

Position	EKP8.5.2	EKP8.5.4	EKP8.7.4
	*δ*_H_ (mult., *J* (Hz))	*δ*_H_ (mult., *J* (Hz))	*δ*_H_ (mult., *J* (Hz))
	-	-	-
3	-	-	6.62 (*s*)
4	-	-	-
5		-	-
6	6.34 (*d*, 2.3)	6.34 (*d*, 2.3)	6.39 (*d*, 2.3)
7	-	-	-
8	6.51 (*d*, 2.3)	6.50 (*d*, 2.3)	6.58 (*d*, 2.3)
9	-	-	-
10	-	-	-
1′	-	-	-
2′	8.06 (*m*)	7.70 (*d*, 7.5)	7.84 (*m*)
3′	7.47 (*m*)	-	6.90 (*d*, 8.8)
4′	7.47 (*m*)	-	-
5′	7.47 (*m*)	6.98 (*d*, 8.8)	6.90 (*d*, 8.8)
6′	8.06 (*m*)	7.70 (*d*, 7.3)	7.84 (*m*)
3-OCH_3_	3.96 (*s*)	3.96 (*s*)	-
5-OCH_3_	3.89 (*s*)	3.96 (*s*)	3.97 (*s*)
7-OCH_3_	3.88 (s)	3.96 (*s*)	3.93 (*s*)
3′-OCH_3_	-	3.90 (*s*)	-
4′-OCH_3_	-	3.87 (*s*)	3.90 (*s*)

**Table 2:** Combination effect of gentamicin and EKP 8.5.2 extract against CRKP.

CRKP	MIC (μg/mL)			FIC of		FICI of	Interpretation
	CN	EKP 8.5.2	CN+ EKP 8.5.2	CN	EKP 8.5.2	CN+ EKP 8.5.2	
CMU001	8	64	2	0.25	0.031	0.281	Synergism
CMU002	8	64	2	0.25	0.031	0.281	Synergism
CMU003	8	64	1	0.125	0.016	0.141	Synergism
CMU004	8	32	2	0.25	0.063	0.313	Synergism
CMU005	8	64	2	0.25	0.031	0.281	Synergism
CMU006	8	128	4	0.5	0.031	0.531	Partial synergy
CMU007	8	64	2	0.25	0.031	0.281	Synergism
CMU008	8	64	2	0.25	0.031	0.281	Synergism
CMU009	8	64	4	0.5	0.063	0.563	Partial synergy
CMU010	8	128	2	0.25	0.016	0.266	Synergism

**Table 3:** Combination effect of gentamicin and EKP 8.5.4 extract against CRKP.

CRKP	MIC (μg/mL)			FIC of		FICI of	Interpretation
	CN	EKP 8.5.4	CN+ EKP 8.5.4	CN	EKP 8.5.4	CN+ EKP 8.5.4	
CMU001	8	64	2	0.25	0.031	0.281	Synergism
CMU002	8	32	2	0.25	0.063	0.313	Synergism
CMU003	8	64	1	0.125	0.016	0.141	Synergism
CMU004	8	32	2	0.25	0.063	0.313	Synergism
CMU005	8	64	2	0.25	0.031	0.281	Synergism
CMU006	8	64	4	0.5	0.063	0.563	Partial synergy
CMU007	8	64	2	0.25	0.031	0.281	Synergism
CMU008	8	64	2	0.25	0.031	0.281	Synergism
CMU009	8	128	2	0.25	0.016	0.266	Synergism
CMU010	8	64	2	0.25	0.031	0.281	Synergism

**Table 4:** Combination effect of gentamicin and EKP 8.7.4 extract against CRKP.

CRKP	MIC (μg/mL)			FIC of		FICI of	Interpretation
	CN	EKP 8.7.4	CN+ EKP 8.7.4	CN	EKP 8.7.4	CN+ EKP 8.7.4	
CMU001	8	64	4	0.5	0.063	0.563	Partial synergy
CMU002	8	32	4	0.5	0.125	0.625	Partial synergy
CMU003	8	64	1	0.125	0.016	0.141	Synergism
CMU004	8	32	2	0.25	0.063	0.313	Synergism
CMU005	8	64	4	0.5	0.063	0.563	Partial synergy
CMU006	8	64	4	0.5	0.063	0.563	Partial synergy
CMU007	8	64	2	0.25	0.031	0.281	Synergism
CMU008	8	64	2	0.25	0.031	0.281	Synergism
CMU009	8	128	4	0.5	0.031	0.531	Partial synergy
CMU010	8	64	2	0.25	0.031	0.281	Synergism

**Table 5:** Combination effect of gentamicin and EKP 8.5.2 extract against CRPA.

CRPA	MIC (μg/mL)			FIC of		FICI of	Interpretation
	CN	EKP 8.5.2	CN+ EKP 8.5.2	CN	EKP 8.5.2	CN+ EKP 8.5.2	
CMU001	8	64	2	0.25	0.031	0.281	Synergism
CMU002	8	32	1	0.125	0.031	0.156	Synergism
CMU003	8	32	4	0.5	0.125	0.625	Partial synergy
CMU004	8	128	2	0.25	0.016	0.266	Synergism
CMU005	8	64	4	0.5	0.063	0.563	Partial synergy
CMU006	8	128	1	0.125	0.008	0.133	Synergism
CMU007	8	128	2	0.25	0.016	0.266	Synergism
CMU008	8	64	4	0.5	0.063	0.563	Partial synergy
CMU009	8	64	2	0.25	0.031	0.281	Synergism
CMU010	8	128	4	0.5	0.031	0.531	Partial synergy

**Table 6:** Combination effect of gentamicin and EKP 8.5.4 extract against CRPA.

CRPA	MIC (μg/mL)			FIC of		FICI of	Interpretation
	CN	EKP 8.5.4	CN+ EKP 8.5.4	CN	EKP 8.5.4	CN+ EKP 8.5.4	
CMU001	8	64	2	0.25	0.031	0.281	Synergism
CMU002	8	64	1	0.125	0.016	0.141	Synergism
CMU003	8	32	2	0.25	0.063	0.313	Synergism
CMU004	8	64	2	0.25	0.031	0.281	Synergism
CMU005	8	64	2	0.25	0.031	0.281	Synergism
CMU006	8	64	1	0.125	0.016	0.141	Synergism
CMU007	8	128	1	0.125	0.008	0.133	Synergism
CMU008	8	64	4	0.5	0.063	0.563	Partial synergy
CMU009	8	32	2	0.25	0.063	0.313	Synergism
CMU010	8	64	2	0.25	0.031	0.281	Synergism

**Table 7:** Combination effect of gentamicin and EKP 8.7.4 extract against CRPA.

CRPA	MIC (μg/mL)			FIC of		FICI of	Interpretation
	CN	EKP 8.7.4	CN+ EKP 8.7.4	CN	EKP 8.7.4	CN+ EKP 8.7.4	
CMU001	8	128	4	0.5	0.031	0.531	Partial synergy
CMU002	8	64	2	0.25	0.031	0.281	Synergism
CMU003	8	64	4	0.5	0.063	0.563	Partial synergy
CMU004	8	64	2	0.25	0.031	0.281	Synergism
CMU005	8	128	2	0.25	0.016	0.266	Synergism
CMU006	8	32	1	0.125	0.031	0.156	Synergism
CMU007	8	64	4	0.5	0.063	0.563	Synergism
CMU008	8	64	4	0.5	0.063	0.563	Partial synergy
CMU009	8	64	4	0.5	0.063	0.563	Partial synergy
CMU010	8	64	2	0.25	0.031	0.281	Synergism

**Table 8:** Combination effect of gentamicin and EKP 8.5.2 extract against CRAB.

CRAB	MIC (μg/mL)			FIC of		FICI of	Interpretation
	CN	EKP 8.5.2	CN+ EKP 8.5.2	CN	EKP 8.5.2	CN+ EKP 8.5.2	
CMU001	8	128	2	0.25	0.016	0.266	Synergism
CMU002	8	64	3	0.375	0.047	0.422	Synergism
CMU003	8	64	2	0.25	0.031	0.281	Synergism
CMU004	8	64	1	0.125	0.016	0.141	Synergism
CMU005	8	64	1	0.125	0.016	0.141	Synergism
CMU006	8	32	2	0.25	0.063	0.313	Synergism
CMU007	8	64	2	0.25	0.031	0.281	Synergism
CMU008	8	64	4	0.5	0.063	0.563	Partial synergy
CMU009	8	64	4	0.5	0.063	0.563	Partial synergy
CMU010	8	64	2	0.25	0.031	0.281	Synergism

**Table 9:** Combination effect of gentamicin and EKP 8.5.4 extract against CRAB.

CRAB	MIC (μg/mL)			FIC of		FICI of	Interpretation
	CN	EKP 8.5.4	CN+ EKP 8.5.4	CN	EKP 8.5.4	CN+ EKP 8.5.4	
CMU001	8	128	2	0.25	0.016	0.266	Synergism
CMU002	8	64	4	0.5	0.063	0.563	Partial synergy
CMU003	8	64	1	0.125	0.016	0.141	Synergism
CMU004	8	64	2	0.25	0.031	0.281	Synergism
CMU005	8	64	4	0.5	0.063	0.563	Partial synergy
CMU006	8	128	1	0.125	0.008	0.133	Synergism
CMU007	8	64	2	0.25	0.031	0.281	Synergism
CMU008	8	128	1	0.125	0.008	0.133	Synergism
CMU009	8	128	2	0.25	0.016	0.266	Synergism
CMU010	8	128	2	0.25	0.016	0.266	Synergism

**Table 10:** Combination effect of gentamicin and EKP 8.7.4 extract against CRAB.

CRAB	MIC (μg/mL)			FIC of		FICI of	Interpretation
	CN	EKP 8.7.4	CN+ EKP 8.7.4	CN	EKP 8.7.4	CN+ EKP 8.7.4	
CMU001	8	128	4	0.5	0.031	0.531	Partial synergy
CMU002	8	64	4	0.5	0.063	0.563	Partial synergy
CMU003	8	64	2	0.25	0.031	0.281	Synergism
CMU004	8	64	4	0.5	0.063	0.563	Partial synergy
CMU005	8	64	2	0.25	0.031	0.281	Synergism
CMU006	8	128	2	0.25	0.016	0.266	Synergism
CMU007	8	64	1	0.125	0.016	0.141	Synergism
CMU008	8	128	2	0.25	0.016	0.266	Synergism
CMU009	8	64	4	0.5	0.063	0.563	Partial synergy
CMU010	8	128	4	0.5	0.031	0.531	Partial synergy

## Data Availability

All data, tables, and figures are original and are available in this article.

## Supplementary Materials

The following supporting information can be downloaded at: https://www.mdpi.com/article/10.3390/plants11223128/s1, Figure S1. NMR analysis of three methoxyflavones isolated from KP. (A) 1H NMR (400 MHz, CDCl3) spectrum of 3,5,7-trimethoxyflavone (EKP 8.5.2), (B) 1H NMR (400 MHz, CDCl3) spectrum of 3,5,7,3′,4′-pentamethoxyflavone (EKP 8.5.4), and (C) 1H NMR (400 MHz, CDCl3) spectrum of 5,7,4′-trimethoxyflavone (EKP 8.7.4).
