# Supplementary material for: Synergistic Effects of Some Methoxyflavones Extracted from Rhizome of Kaempferia parviflora Combined with Gentamicin against Carbapenem-Resistant Strains of Klebsiella pneumoniae, Pseudomonas aeruginosa, and Acinetobacter baumannii
Source: Plants (Basel). 2022 Nov 16;11(22):3128. doi: 10.3390/plants11223128 (PMC9695190; doi:10.3390/plants11223128)
Supplement: Supplementary file 1 [file plants-11-03128-s001.zip › plants-1985891-supplementary.pdf]

Supplementary Materials

(A)

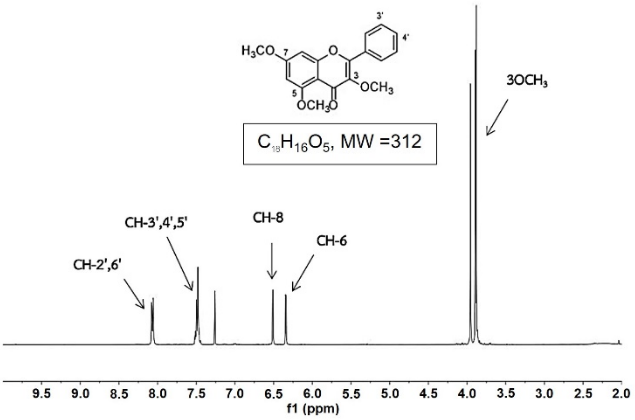

(B)

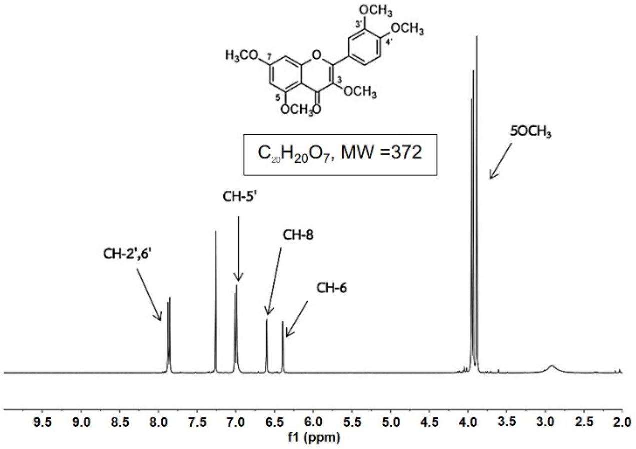

(C)

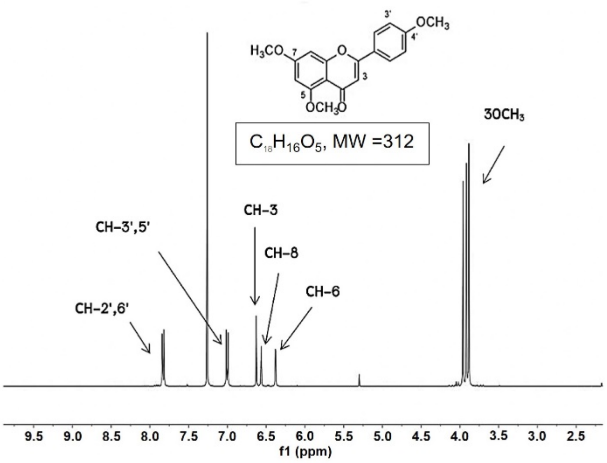

Figure S1. NMR analysis of three methoxyflavones isolated from KP. (**A**) 1H NMR (400 MHz, CDCl3) spectrum of 3,5,7-trimethoxyflavone (EKP 8.5.2), (**B**) 1H NMR (400 MHz, CDCl3) spectrum of 3,5,7,3′,4′-pentamethoxyflavone (EKP 8.5.4), and (**C**) 1H NMR (400 MHz, CDCl3) spectrum of 5,7,4′-trimethoxyflavone (EKP 8.7.4).
